# The Population-Level Surveillance of Childhood and Adolescent Cancer and Its Late Effects in Europe with an Example of an Effective System at the Slovenian Cancer Registry †

**DOI:** 10.3390/cancers17040580

**Published:** 2025-02-08

**Authors:** Ana Mihor, Carmen Martos, Francesco Giusti, Lorna Zadravec-Zaletel, Sonja Tomšič, Katarina Lokar, Tina Žagar, Mojca Birk, Nika Bric, Vesna Zadnik

**Affiliations:** 1 Slovenian Cancer Registry, Institute of Oncology Ljubljana, 1000 Ljubljana, Slovenia; stomsic@onko-i.si (S.T.); klokar@onko-i.si (K.L.); tzagar@onko-i.si (T.Ž.); mbirk@onko-i.si (M.B.); nbric@onko-i.si (N.B.); vzadnik@onko-i.si (V.Z.); 2 Medical Faculty, University of Ljubljana, 1000 Ljubljana, Slovenia; 3 European Commission, Directorate General Joint Research Centre (JRC), 21027 Ispra, VA, Italy; francescogiusti@hotmail.com; 4 Foundation for the Promotion of Health and Biomedical Research in the Valencian Region (FISABIO), 46020 Valencia, Spain; carmen.martos@fisabio.es; 5 Department for Radiotherapy, Institute of Oncology Ljubljana, 1000 Ljubljana, Slovenia; lzaletel@onko-i.si

**Keywords:** childhood and adolescent cancer, survivors, late effects, population-level surveillance, cancer registry

## Abstract

Background: The registry-based collection of detailed cancer and late effect (LE) data in childhood and adolescent cancer (CAC) is rarely explored. Aim: We aimed to provide an overview of CAC registration practices in Europe and share a Slovenian example. Methods: We distributed a questionnaire among European cancer registries on disease, treatment and LE registration and present the system at the Slovenian Cancer Registry along with an example of retrospectively collected LE data from a cohort of central nervous system tumour survivors from 1983 to 2000. Kaplan–Meier and Cox regression were used to calculate the LE incidence. Results: Out of 27 responding registries, over 80% registered cancer type, vital status, death and second primary cancer data. Less than 20% registered cumulative doses of radiation and systemic therapy or progressions. Only three registered LEs. The obstacles in setting up LE collection in registries are a lack of standardization in the variable sets, definitions and methods of collection. In the retrospective cohort, neurological and endocrine LEs were most common. Females had a higher risk of endocrine LEs (HR of 1.89; 95% CI of 1.08–3.31), while patients treated with radiotherapy had higher risks of endocrine (3.47; 1.80–6.69), musculoskeletal and skin LEs (3.16; 1.60–6.26) and second primary cancers (2.85; 1.18–6.75). Conclusions: Standardization and harmonization are necessary to promote detailed CAC and LE registration.

## Simple Summary

The collection of detailed data, including on diseases that develop as a result of treatment later in life (i.e., late effects), on children and adolescents with cancer through cancer registries is important to provide exact, complete and clinically relevant results. Therefore, we surveyed European registries about their practices, revealing that collecting data on late effects is not widespread and that most registries collect only a basic set of data with little clinical detail. The approach of the Slovenian Cancer Registry is also presented, along with observations obtained through the setting up and testing processes, namely the lack of standardization in the variable sets, definitions and methods of collection and the poorer quality of results with retrospective registration. This research highlights the need for stronger collaboration between registries in order to harmonize collection practices, as this would facilitate the collection of more than just a basic dataset by population-based cancer registries.

## 1. Introduction

Cancer in childhood and adolescence (defined here as occurring under the age of 20) has become a highly curable disease due to improvements in treatment [1,2,3]. While some data trends indicate a potential rise in the incidence of certain types of childhood and adolescent cancer, survival is improving and the mortality is decreasing in Europe and other high-income countries [4,5], leading to a growing and ageing cohort of childhood and adolescent cancer survivors (CACSs). However, this success comes with a burden of late sequelae in long-term survivors on account of the toxic effects of treatment on healthy tissues as well, especially as patients receive treatment while they are still growing and developing [6]. Additionally, there are reports of accelerated ageing in survivors as a pathological mechanism underlying some of the observed excess morbidity and mortality [7,8]. Large cohort studies [9,10,11] have provided a wealth of data on the type and frequency of late effects in survivors, such that overall follow-up guidelines [12,13] and specific internationally harmonized guidelines have been developed for the surveillance of breast cancer [14], cardiomyopathy [15], ototoxicity [16], premature ovarian insufficiency [17], gonadotoxicity [18] and thyroid cancer [19].

On the other hand, there are scarce data on the surveillance of late effects as part of population-based cancer registry (PBCR) activities. PBCRs have a rich history of continuous, systematic and harmonized data collection practices for the monitoring of standard cancer burden indicators [20]. While the primary aim of PBCRs is the complete registration of high-quality data, it is equally important to harmonize data across different countries. The European Network of Cancer Registries (ENCR) is an important institution in efforts to standardize datasets on an international level with the aim of better comparability and increased possibilities of pooled data analyses [21]. The standardization of practices in the estimation of the cancer incidence burden is at a high level, whereas the field of the late effects of cancer has not yet been improved in this regard. Having the necessary knowledge, technology, legal basis and other resources, PBCRs are the most appropriate setting for the prospective and routine monitoring of the population burden of late effects, which would bring several advantages in terms of complete estimates of the burden as well as more accurate time trends and the health system resources needed for its management. For late effects surveillance, very detailed treatment data are of the utmost importance. These are also vital in view of efforts to introduce a common European Survivorship Passport [22,23].

The first aim of our research was to provide an overview and comparison of population-level registration practices for childhood and adolescent cancers and the late sequelae of their treatment in Europe. Further, we present the effective system recently activated in the population-based Slovenian Cancer Registry (SCR): the structure of the database, along with the dataflow, is given for the newly established Slovenian Childhood Cancer Clinical Registry (SCCCR). Slovenia is a middle European country with one of the most developed cancer registration systems globally [19]. The establishment of the SCCCR was envisioned by the National Cancer Control Programme of Slovenia, with the aim of improving knowledge on the quality of treatment as well as detailed outcomes in terms of late effects. For the illustration of the possible outputs of the SCCCR, an analysis of the late effects in a cohort of childhood and adolescent central nervous system cancer patients was prepared.

## 2. Materials and Methods

First, to map cancer registry practices, we sent out an online questionnaire-based survey to members of the ENCR. The survey was open to the members from 1 July 2022 to 31 August 2022 via the EUSurvey tool, emailed directly to 140 members (the majority of which, around 70%, were regional, and 30% were national) and advertised in the ENCR Newsflash for July 2022 with an additional reminder to boost the response rate. The complete questionnaire is available in Appendix A. It was divided into several sections, the first on the coverage of the registry (national/regional, the age range of pediatric patients, the malignant and non-malignant entities included), the second on whether PBCRs collect or are planning to collect detailed data (on the diagnosis, staging, treatment, progression, vital status follow-ups and other data) and the third on whether they register late effects data or are planning to, as well as how these data are or are going to be collected and coded. The survey results were analyzed using descriptive statistics; namely, we categorized questions addressing the availability of variables into categories (diagnosis, staging, follow-up, primary systemic therapy, surgery and radiotherapy) and reported the number and percentage of registries who answered either “yes”, “no” or “planning to” to questions from each category regarding the collection of specific variables. Furthermore, individual registries were ranked according to their category and overall summary scores, which were calculated by summing answer-specific scores (0 points were allocated to answers of “no”, 1 point for answers of “planning to” and 2 points for answers of “yes”). The data were presented in stacked bars and heat maps.

Second, we describe the elements of the SCCCR, the variable set, the sources of information and the methods of collection, in text and diagrams. For the illustration of the possible outputs of the SCCCR, an analysis of the late effects in the tested cancer patients’ cohort was prepared. Adults older than 19 years with childhood and adolescent central nervous system (CNS) tumours were included in the testing cohort. CNS tumour patients were selected to ensure a large enough number of patients with a high number of expected events (late effects). We extracted from the SCR all cases of cancers with an ICD-O-3 topography of C70.0–C72.9 who were diagnosed with a first primary cancer at the age of 0–19 years between 1983 and 2000. We also extracted all second primary tumours (all malignant cases as well as melanoma in situ—ICD-10 D03 and non-malignant CNS tumours—as defined above) in these cases. We excluded patients who died before the age of 20, as well as those who died due to the protracted course of their primary tumour. By examining electronic patient files held at the Institute of Oncology Ljubljana, we then determined which patients had received a late sequelae follow-up at the Late Effects Clinic, which is a centralized service with national coverage for the surveillance of CACSs. We extracted information on late effects (as recorded by the end of February 2023), which we defined as early-onset but long-term or late-onset somatic health events. We adapted the Common Terminology Criteria for Adverse Events (CTCAE)-based classification developed by Hudson et al. [24] and classified late effects into organ systems (Table S2), recording the most accurate known date of onset of the first late effect according to the organ systems, as well as the number of all different known late effects recorded according to the by organ systems.

One of the major problems in preparing the classification was that we had difficulties in adapting it for use by Late Effects Clinic physicians as well as for cancer registries’ purposes, where it might not be feasible to code every late effect to the highest level of detail. Therefore, unlike Hudson et al. [24], we included surgical procedures, because if the grading of organ failure was not possible, registering gross surgical procedures was deemed important. Similarly, some other surgery-related sequelae were also considered important, such as tissue defects, etc. Furthermore, we were unsure as to what detail certain events should be recorded with (for example, any valve defect or a specific valve defect) as well as how to include broader categories, if details were not available. We resolved this by adding broader and unspecific categories (not listed in the classification). During testing, we came across several barriers. As expected, grading was not possible to determine retroactively, because the level of detail in the electronic files was too low. The accuracy of dates was also poorer because of the retrospective approach. Furthermore, it was difficult to ascertain in which cases a medical condition was truly the result of cancer and its treatment; therefore, we collected all newly developed medical conditions recorded by the Late Effects Clinic. Additionally, since no specific classification existed, physicians were not systematically describing medical conditions in electronic patient files as late effects and the level of detail varied. Neurocognitive late effects in particular were often described only vaguely. Nevertheless, it was possible to at least count the number of different late effects (as classified in Table S2) according to the organ systems and record the most accurate known incidence date for each organ system.

The cohort characteristics were presented using descriptive methods. We determined the prevalence of late effects at the last follow-up in the Late Effects Clinic (as of the end of February 2023). In SPSS, Version 29.0, we calculated the cumulative incidence using the Kaplan–Meier method to ascertain the probabilities of developing second primary tumours and organ-specific late effects (cardiovascular, respiratory, gastrointestinal and hepatobiliary, renal and urinary, reproductive tract, endocrine, musculoskeletal and skin, neurological and neurocognitive, immunological and infectious, hematological, auditory, and visual). Psychosocial effects were not included. The time to event was defined as the time from the date of diagnosis to the date of onset of the first organ-specific late effect or the date of diagnosis of a second primary tumour. Patients were censored at the last known date of follow-up at the Late Effects Clinic (or 6 February 2023 for second tumour analysis). Patients without a known follow-up (those not invited/not responding to follow-up invitations) had zero follow-up time. Furthermore, we used Cox regression analyses to analyze the cumulative incidence of all late effects combined as well as late effects grouped according to the organ systems with the sex, the type of anticancer treatment received (surgery, yes/no; radiotherapy, yes/no; systemic therapy, yes/no) and the period (1983–1990 and 1991–2000) as covariables with significance at a *p*-value of less than 0.05.

## 3. Results

### 3.1. Survey on Cancer Registry Practices in Field of Late Effects of Childhood and Adolescent Cancers

We received responses from 27 European cancer registries (response rate of 19.3%; Table S1). Of these, there were 12 with national (Belgium, Croatia, Estonia, the Faroe Islands, Finland, Ireland, Luxembourg, Montenegro, Romania, Slovenia, Switzerland and the Netherlands) and 15 with regional coverage (from England, Germany, Italy, Romania and Spain). All in all, 16 different European countries provided responses. Six of the responding registries were childhood registries; others covered all ages. We searched online information on the registration systems of the non-respondents deemed important for a better outline of the practices (especially childhood cancer-dedicated registries), which is presented in the discussion.

Regarding individual registries, according to the overall summary score, the top three were the Belgian and Swiss registries and the West Midlands tumour registry, United Kingdom (Table S1). Though a formal comparison of national versus regional registries was not feasible due to insufficient numbers, the results showed that among ten registries with the highest overall scores, two were national, while this number was only two among the bottom ten registries. All PBCRs registered all malignancies (i.e., they were not specific for, e.g., hematologic malignancies only). Only two registries (the Faroese Cancer Registry and the Cancer Registry of Friuli Venezia Giulia) did not and were not planning to register any non-malignant childhood and adolescent tumours. Of the remaining 25 registries, 24 already registered, at least to some degree, (and 1 was planning to) the non-malignant International Classification of Diseases for Oncology, 3rd Edition [25] (ICD-O-3), codes C70–C72 (meninges, brain and spinal cord, cranial nerves and other parts of the CNS), whereas 19 registered (3 were planning to) ICD-O-3 codes C75.1–C75.3 (pituitary gland, craniopharyngeal duct and pineal gland). The National Cancer Registry Ireland also registered in situ and uncertain behaviour tumours for all sites and the Belgian Cancer Registry all in situ locations, while several others included a wide variety of locations for in situ and uncertain behaviour tumours, most commonly bladder, ovary and skin melanoma.

**Figure 1:**
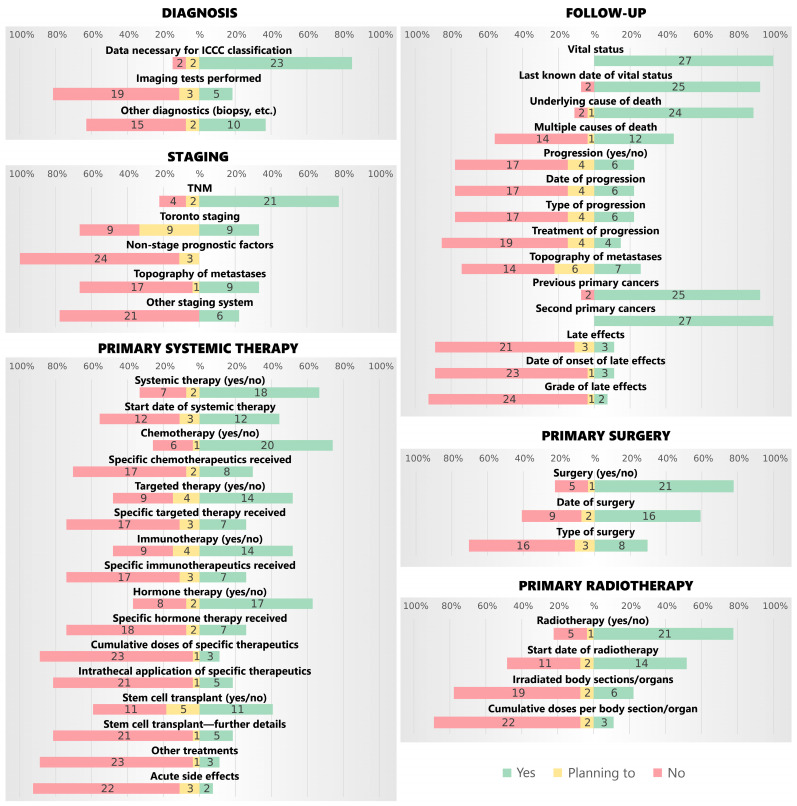
Number and percentage of all answers (n = 27) on data availability in cancer registries by type of data.

Figure 1 presents the questionnaire results for the availability of data on the diagnosis, staging, therapy and follow-up. A high percentage of respondents (over 80%) collected data to classify childhood and adolescent tumours according to the International Childhood Cancer Classification (ICCC), third edition [26], and data on the vital status, the underlying cause of death and other primary tumours. On the other hand, few (less than 20%) registered the imaging diagnostics performed, cumulative doses of therapeutics, cumulative radiation doses, details of stem cell transplants, other treatments received and acute side effects of treatment, as well as data on the progression of the disease. Only three registries responded that they registered late effects (the Swiss Childhood Cancer Registry, the Belgian Cancer Registry and the West Midlands Regional Children’s Tumour Registry), and only three expressed intent to introduce it (the Girona Cancer Registry, Romanian National Childhood Cancer Registry and the SCCCR of the Slovenian Cancer Registry). No responding registry currently collected non-stage prognostic indicators. Other items had between a 20 and 80% availability, with a notably high percent of the intention to introduce Toronto staging and expand the detail on systemic therapy and disease progression.

### 3.2. Development and Features of Slovenian Childhood Cancer Clinical Registry

**Figure 2:**
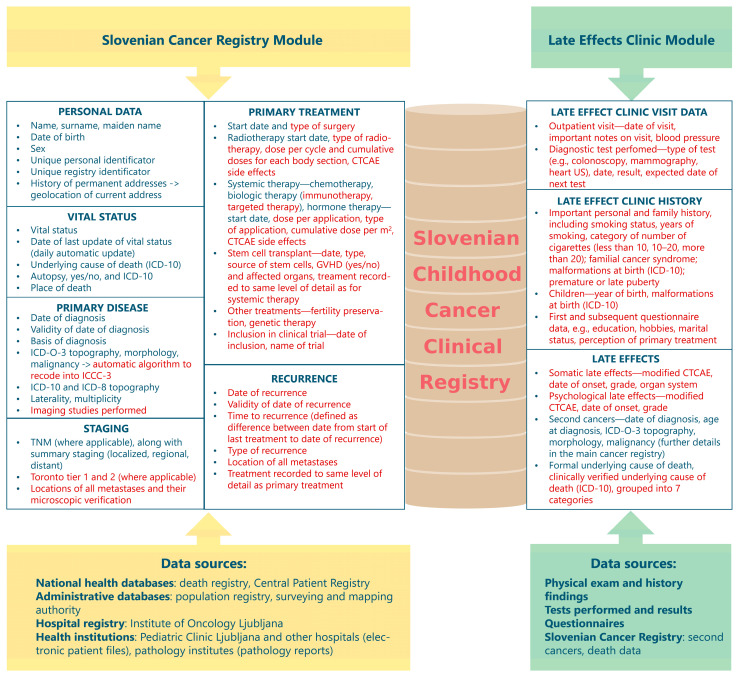
Data sources and variables of the Slovenian Childhood Cancer Clinical Registry (SCCCR). Red items represent the expanded set of variables in the SCCCR, whereas blue items were already in place in the Slovenian Cancer Registry before the introduction of the SCCCR.

The SCCCR was developed as an extension of the population-based SCR, with the collaboration of SCR staff, physicians working at the Late Effects Clinic and the Pediatric Clinic Ljubljana and IT experts. We also drew on good practice examples from abroad, such as the New Zealand Children’s Cancer Registry and the Belgian Cancer Registry. It has in place the same internationally harmonized practices as the SCR, such as international coding rules, active registration, linkage with administrative databases and data protection measures, while at the same time building on these practices to vastly expand the number of collected variables (see Figure 2 to compare the SCR and the SCCCR variable sets).

The SCR and SCCCR operate through two-way communication. All diseases registered within the special SCCCR platform are automatically transferred into the SCR (only the basic set of data on the disease, staging and treatment), and after the disease is also registered in the SCR, certain data available there are then transferred from the SCR to the SCCCR (such as personal data, the vital status, death data). Because the SCR is linked to the population registry, the personal data and vital status are very accurate and updated every 24 h.

The population covered by the SCCCR is all persons residing in Slovenia who were aged 0–19 years at diagnosis. We collect all malignant ICD-10 codes and non-malignant intracranial and intraspinal tumours (ICD-10 D32–D33, D35.2–D35.4, D42–D43, D44.3–D44.5), as well as certain other non-malignant codes (D03, D05, D06, D09.0, D39.1).

The SCCCR has two modules: (1) Since the primary aim was to create a platform that would enable the systematic registering and long-term monitoring of late effects (in terms of morbidity and mortality) in a population of CACSs, one module was designed for direct use by clinicians at the Late Effects Clinic at the Institute of Oncology Ljubljana, where entry is possible either retrospectively or prospectively, allowing them to access all historical data during patients’ visits and the ongoing collection of clinical data from exams, questionnaires and other follow-up findings. This module does not have 100% population coverage, as patients eligible for follow-up are those treated for tumours at the Pediatric Clinic (over 80% of all cases aged 0–14 years, fewer adolescents), aged 18 years or more and at least three years post treatment, but the coverage for the eligible population is almost complete. (2) For the proper evaluation of the burden of late effects in survivors, data on the primary cancer and treatment received are also of vital importance. Therefore, the secondary aim was to collect as much detailed data on disease and treatment as possible through a module managed by specially trained SCR staff, who extract the data manually directly from electronic patient records held at the Pediatric Clinic at the University Medical Centre Ljubljana, which treats the vast majority of children and adolescents with cancer, as well as other treating institutions. This module covers the whole childhood and adolescent cancer patient population and started collecting data since the incidence year of 2019. Of note, the cumulative doses per m^2^ of the body surface of systemic therapy and cumulative radiation doses per organ are collected. This allows for analyses of the quality of treatment, as well as the short- or long-term outcomes.

### 3.3. Late Effects in Childhood and Adolescent Central Nervous System Cancer Patients

For the illustration of the possible outputs of the SCCCR, an analysis of the late effects in a cohort of childhood and adolescent central nervous system cancer patients was prepared. There were 240 cases of childhood and adolescent CNS tumours (0–19 years) diagnosed between 1983 and 2000. After eliminating cases who died before adulthood or due to protracted primary disease in early adulthood (Figure S1), there were 126 CACSs who were eligible for a late effects follow-up. Of those, the majority were male, and there were no observed differences between sexes in the age at diagnosis, malignancy, period of diagnosis, treatment and follow-up (Table 1). Of note, around 25% of eligible patients (32 out of 126) never received a late effects follow-up at the Late Effects Clinic. At least one late effect was observed in more than 90% of patients followed at the Late Effects Clinic. At the last known follow-up date for late effects, the prevalence was highest for neurologic (73.4%), endocrine (57.4%) and musculoskeletal and skin late effects (55.3%).

**Figure 3:**
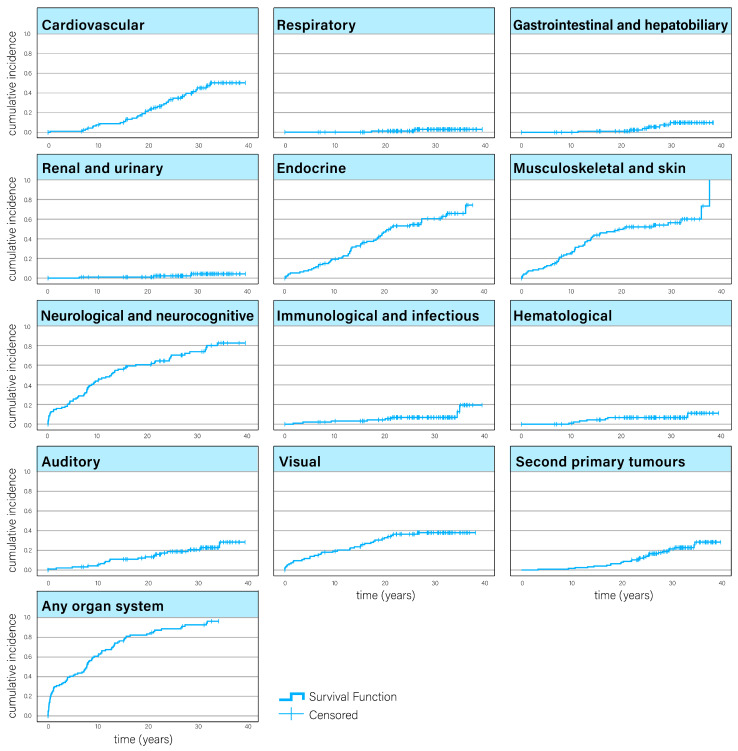
Cumulative incidence curves for somatic late effects and second primary cancers.

The results of Kaplan–Meier analyses (Figure 3) revealed that CACSs with CNS tumours in the cohort were at risk for developing a multitude of organ-specific late effects (with the exception of reproductive late effects, of which none were registered) and second primary tumours long after their primary treatment had finished, though, in general, there was a levelling off in the cumulative curves after around 20–30 years. For endocrine late effects in particular, the cumulative incidence curve did not level off.

The results of Cox regression analyses by the organ system and for second primary tumours are in Table S3. We report only on those organ systems with an event number of more than 10. In brief, females seemed to be at higher risk for endocrine late effects (HR of 1.89, CI of 1.08–3.31), while radiotherapy was a risk factor for endocrine (HR of 3.47, CI of 1.80–6.69) and musculoskeletal and skin late effects (HR of 3.16, CI of 1.60–6.26) as well as second primary cancers (HR of 2.85, CI of 1.18–6.75). The risk of second primary tumours did not differ among sexes. There was a higher risk for neurologic and neurocognitive effects in the later period (1991–2000; HR of 2.18, CI of 1.31–3.62). Although not significant, systemic therapy seemed associated with the risk of cardiovascular events (HR of 2.05, CI of 0.96–4.37).

## 4. Discussion

### 4.1. Existing Practices in Pediatric Cancer and Late Effects Registration in Europe

With the use of the questionnaire, we sought to elicit information on current and possible future data collection systems among ENCR members. The survey results, although not comprehensive due to the relatively low number of respondents, provide an insight into which kinds of pediatric cancer data are available in Europe and to what degree. It seems that, according to the overall score, more national registries were higher ranked compared to regional ones, which might reflect differences in the available resources. Our primary aim was to evaluate late effects surveillance, and the results are not encouraging, since a very small percentage reported this type of monitoring or an intent to introduce it, and one of those was Slovenia. A low number of PBCRs also collected more than basic (yes/no for the main modalities) treatment data. Detailed treatment data are vital when investigating long-term outcomes. The only areas with good coverage were the vital status and second primary tumours, which all general PBCRs already collected. Thus, the only possibility for widespread analyses of late effects using PBCR data are the mortality and incidence of second primary cancers. These robust indicators are of value and should be used more, especially in a standardized, internationally comparable fashion, such as within the CRICCS project for second primary tumours [27]. Nevertheless, they are insufficient for any in-depth studies looking into survivorship long-term health trends. A noteably high percentage of respondents already had or were planning to introduce Toronto staging. This is no doubt due to the good example of harmonization efforts within the BENCHISTA project, which aims to standardize and promote the collection of the endorsed registry-adapted pediatric-specific staging guidelines across the world [28,29].

Of the participating registries, three already had late effects registration in place. The Swiss Childhood Cancer Registry [30] had been collecting detailed data on treatment and outcomes (such as recurrences, late effects and second primary tumours) from treating institutions in yearly intervals. The late effects were classified according to the ICD-10 classification, meaning the grading was not recorded. The once-yearly update is also not optimal, although the data could still be very accurate if they are collected prospectively at the treating institutions. The Belgian Cancer Registry has been collecting data on late effects classified according to the CTCAE from hospital databases since 2004. Additionally, since 2017, the Belgian Cancer Registry has run an affiliated project called “Pediatrics—Late effects”. The data are collected via online registration forms by the treating physicians, who send one primary and several follow-up registration forms after progression/relapse or a new primary tumour, as well as regularly every 5 years after diagnosis. Here, again, the 5-yearly update might be a small disadvantage in terms of the data availability and/or accuracy. The West Midlands Regional Children’s Tumour Registry has been using CTCAE-based late effects registration, complete with grading, since 1997. It is part of an oncology department with regional coverage. Their data sources are hospital databases (the follow-up clinic), outpatient databases and primary health care institutions, which are actively contacted for data on patients not participating in the hospital follow-up [31].

A literature search for additional information on practices revealed a few further examples (Table S4). In France, the National Childhood Cancer Registry collects both detailed disease and treatment data. They also collect late effects data in a passive way from medical databases. Additionally, in 2013, they launched the French Childhood Cancer Observation Platform [32], which has data on all childhood cancers under 15 years. The platform receives core basic data from the national childhood registry, while also including geolocation and biobank data and collecting data on treatments (including cumulative doses) and late effects (relapses and second cancers in childhood) retrospectively every 5 years from medical records, linking yearly to the national health insurance information system (for morbidity, disabilities and care consumption) and adding online questionnaire data from childhood cancer survivors (CCSs). It is a good example of compiling diverse data from many sources through linkage as well, though a disadvantage is that the late effects are not ascertained clinically. The German Childhood Cancer Registry, while a PBCR, also has features of a clinical registry, receiving stage and follow-up data from clinical studies for over 40,000 CCSs [33,34]. Like the French example, it is well set up for the population-based surveillance of late effects, though it lacks therapy details because it has no direct access to hospital records. Having input from different clinical studies with different designs and inclusion criteria might also preclude it from recording late effects in a standardized way for all CCSs. In Greece, the Nationwide Registry of Childhood Hematological Malignancies and Solid Tumors (NARECHEM-ST), in addition to basic disease data, collects information on death, relapse and bone marrow transplantations, as well as long-term sequelae, though the latter have not yet been sufficiently systematized [35]. The Hungarian Childhood Cancer Registry also collects some detailed data on treatment and disease as well as late effects, but the variable set and whether registration is systematic and continuous were hard to ascertain [36]. The regional Yorkshire Specialist Register of Cancer in Children and Young People [37] employs many modern data extraction and linkage practices with a variety of sources, including detailed treatment and late-outcome data (secondary care admissions). Of note, they include young adults and link with the National Pupil Database to obtain educational data. The Spanish Registry of Childhood Tumors (RETI-SEHOP) is a national registry compiling regional data primarily to investigate the incidence, causes of cancer and survival in Spain, whereas late effects do not seem to be prominently featured [38]. In southern Sweden [39], a similar approach to the one in Slovenia was undertaken in order to provide clinicians with reliable and complete PBCR data with the aim to facilitate efficient and appropriate medical follow-ups. Their PBCR, the clinical childhood cancer registry (BORISS) [40], although not part of the national PBCR, includes patients diagnosed at 0–18 years old from 1970 onwards and has detailed treatment data on 5-year survivors, retrospectively collected until 2016 and prospectively since. Furthermore, they annually import certain core data from the Swedish national PBCR as well as the national population registry. BORISS provides the late effects clinic in Skåne with a treatment summary for tailored surveillance. The Dutch Late Effects Registry (LATER) [41], which is also not part of a PBCR, is population-based as its design was based on an information model implemented in all Dutch oncology centres. It has a long tradition of use, and many studies have come about since its introduction, though it seems the studies are based on a closed cohort [42]. The PBCR approach, on the other hand, assumes an open cohort approach. The Swiss Young Survivors at KSA is another example of a registry not affiliated with a PBCR that has a very similar design to how the SCCCR was set up [43]. They collect detailed data on disease and treatment and late effects through follow-up visits, using Hudson et al.’s modified CTCAE classification [24]. Their advantage is that they start collecting follow-up data in a prospective manner immediately after patients enter follow-up care while still in childhood and adolescence, which closes the gap between diagnosis and adulthood. Furthermore, they register detailed data on physical, laboratory and imaging evaluations and other tests, which would allow for assessing the agreement between different assessors or reclassification using other classifications. They have retrospective data from 2016 to 2021 and prospective data onwards.

The main limitation related to the survey was the low response rate, though we endeavoured to overcome this by searching for good practice examples among childhood cancer-dedicated ENCR members. Nevertheless, important PBCRs were perhaps missed. Since we only contacted ENCR members, non-PBCR registries (who are also not part of the ENCR) that could have provided valuable information for PBCR-based surveillance were excluded. In searching for additional examples, we could have made mistakes in the interpretation of online findings as well.

### 4.2. Barriers to Registering Detailed Childhood Cancer Treatment and Outcomes

The main barriers to registration in PBCRs are likely similar to those that PBCRs usually encounter in their work—a lack of access to data (legal barriers, technical barriers, low-quality or incomplete data sources, no established follow-up services, etc.), a lack of human and financial resources and a lack of awareness of the importance of such surveillance, as well as methodological barriers. Specifically, access to electronic patient files through active registration is of paramount importance to be able to access high-resolution data, which is hard for many registries. A vital aspect is also the age definition for childhood and adolescent cancer, as it varies among treating institutions and registries. Pediatric institutions across Europe have different upper age limits for the cancer patients they treat, which could affect registration as well [44]. The standardization of cancer treatment and recurrence coding is also lacking for now, but this might soon be made available through dedicated ENCR working groups [45]. In this regard, it would be important to also document any treatment deviations from established protocols, as these can be significant factors in survival and other outcomes [46]. Another important barrier to the standardized registration of late effects is that currently, the methods for assessing and defining long-term outcomes in childhood cancer survivors are not harmonized. A systematic review by Streefkerk et al. [47] found a large variability in the way researchers ascertain and classify health outcomes in survivors, stressing that we need global harmonization initiatives to improve survivorship research. Cancer registries in particular need to contribute to these efforts in order to achieve the same comparability and consistency level as that of standard cancer registry datasets. In order to improve the quality of cancer registry data on pediatric cancer treatment and outcomes, PBCRs need to form a data exchange agreement with clinical institutions or national/regional patient database administrative systems. A recent feasibility study from Switzerland [48] on the prospective registration of late effects by treating physicians used the modified CTCAE developed by Hudson et al. [24], and its results speak in favour of this type of approach to monitoring the late effects burden. The authors stress the importance of standardization in collection and confirm that this type of monitoring is feasible and provides a rich database for clinical as well as research purposes. During the development and testing of the SCCCR, we reached similar conclusions, which we describe below.

### 4.3. The Lessons Learned from the Slovenian Example

While we are aware that the example of Slovenia, a country of only 2 million inhabitants, might not be useful for countries that have vastly different legislation, management of CACSs (especially in terms of smaller countries with centralized services versus larger countries with many centres and registries), possibilities for the interinstitutional exchange of data or other established registry practices, it is important for registries to share practices and experiences. The main advantage of the Slovenian example is the exchange and enrichment of data between three institutions with national coverage: the Pediatric Clinic, SCR, and Late Effects Clinic. The SCCCR deepens the level of registry data, making them more useful for clinically important analyses, while also providing a platform to improve the follow-up at the Late Effects Clinic. The lack of the harmonized classification of late effects was a significant barrier, though, and we propose that it should entail several tiers based on the availability of clinical details to at least enable the registration of broader categories with suggestions for grading. We plan to promote this idea within the ENCR and International Association of Cancer Registries (IACR) community through conferences [49], expert meetings and international projects.

Through the exploratory study, we found that, at least in Slovenia, a retrospective approach based on searching for information in electronic medical files is not ideal and has many disadvantages in terms of the data quality, depth and completeness compared to prospective entries by treating physicians, which might be a better way for PBCRs to access very clinically complex data. Nevertheless, the CSN CACSs cohort late effects results were in line with many previous studies on late effects. We found a high prevalence of all previously reported common chronic health conditions, such as endocrine, neurological and neurocognitive sequelae and second primary tumours [50,51,52,53]. The higher risk of endocrine, musculoskeletal and skin sequelae and second tumours in irradiated patients is in line with previous studies which guidelines have been based on [12,13,54]. The relevance of the higher risk of endocrine events in women and the higher risk of neurological and neurocognitive effects in the later period are difficult to ascertain. Given that treatment has improved in terms of toxicity and that neurocognitive effects can often manifest only years after treatment, we would have expected a reverse outcome. It is perhaps a phenomenon following temporal changes in the practices of registration by physicians or an effect of more patients with CNS tumours with a previously poor prognosis surviving recently due to improved treatment, while the finding of a higher risk of endocrine effects in women is in line with previous studies [55]. An additional finding from this study was the relatively high percentage of presumably eligible patients who never received a follow-up. This was attributed partly to patients “falling through the cracks” of the current referral system, where the Pediatric Clinic is the main referring institution and therefore patients treated there are more likely to be referred to an adult follow-up, but is in large part consequent to patients opting out. Having these types of data is useful in evaluations of the current follow-up systems.

### 4.4. Possible Advantages of Harmonization in Childhood Cancer Disease and Outcome Registration

As important as large childhood cohort studies are, introducing the systematic continuous and standardized surveillance of late effects within or as part of PBCRs has certain advantages. It would allow for more complete cancer burden estimates and determine the percentage of eligible patients receiving an appropriate follow-up. Compiling and endorsing a late effects classification would stimulate and improve the reporting on late effects by treating physicians, since their practices might differ between institutions, countries and across time. Psychosocial outcomes in particular are, though very impactful, unsystematically documented in our experience; therefore, we were unable to include them in this exploratory study of CNS CACSs. Harmonization would also improve the PBCR data quality in many countries and aid in pooling data across European countries, a useful approach in studying rare cancers, and provide more opportunities for high-resolution survivorship studies. Better data on cancer and outcomes could serve as a basis for advancing the initiative for the “right to be forgotten”, namely that survivors face unjust financial obstacles, such as access to loans, after they have been cured of cancer, which has been increasingly recognized across Europe [56]. Detailed treatment data are also important with respect to international initiatives such as the Survivorship Passport (SurPass) [22], the aim of which is to provide a standardized childhood cancer treatment summary in Europe. Until now, the first version has been piloted in Italy, but its design is considered too time-consuming as each data item needs to be manually entered by hospital staff. Therefore, a new version is under development which will allow for semi-automatic data entry and has IT tools integrated for the automatic extraction of certain data, if they are available. Initially, six European countries will test the platform. The major barriers to this version of the SurPass [23] are access to clinical data sources and data harmonization between different institutions, such as hospitals and cancer registries, each of which hold data relevant for the SurPass. Therefore, embedding clinical registries within PBCRs offers a unique opportunity for enabling the adoption of the SurPass, as such registries have already addressed the most important obstacles by forming collaborations with clinicians and exchanging data between relevant institutions and can provide nationally relevant treatment reports to improve and tailor surveillance.

## 5. Conclusions

In order to improve the data quality and detail in pediatric cancer registration, access to electronic clinical patient data is an important option for cancer registries to explore within the data availability and protection restraints. Applying the PBCR approach to clinically relevant data will lead to improved childhood and adolescent cancer burden estimates and trends, while allowing for analyses of risk stratification approaches for follow-ups. PBCRs should proactively seek for whether there are possibilities for such interinstitutional collaboration and most importantly collaborate at the European and international levels to develop harmonized treatment and late effects registration practices as well as publish examples of good practices. Recently, the most prominent experts in the field of childhood cancer and cancer registration held an event in Paris in 2023, where the case for the International Childhood Cancer Data Partnership was outlined along with important obstacles, including harmonization and standardization [57].

## Figures and Tables

**Table 1:** Characteristics of the childhood and adolescent central nervous system tumour survivor cohort. SD—standard deviation; n—number; y—years.

Sex, n (%)	Both Sexes	Male	Female
**Characteristic**	n = 126 (100.0)	n = 79 (62.7)	n = 47 (37.3)
**Age at diagnosis (y), mean ± SD**	10.5 ± 5.3	10.4 ± 5.5	10.7 ± 5.0
**Age at diagnosis group (y), n (%)**			
0–4	26 (20.6)	18 (22.8)	8 (17.0)
5–9	34 (27.0)	20 (25.3)	14 (29.8)
10–14	33 (26.2)	21 (26.6)	12 (25.5)
15–19	33 (26.2)	20 (25.3)	13 (27.7)
**ICD-10 code, n (%)**			
C	99 (78.6)	65 (82.3)	34 (72.3)
D	27 (21.4)	14 (17.7)	13 (27.7)
**Diagnosis period, n (%)**			
1983–1990	61 (48.4)	38 (48.1)	23 (48.9)
1991–2000	65 (51.6)	41 (51.9)	24 (51.1)
Treatment, n (%)			
Surgery, yes; n (%)	111 (88.1)	72 (91.1)	39 (83.0)
Radiotherapy, yes; n (%)	56 (44.4)	34 (43.0)	22 (46.8)
Systemic therapy, yes; n (%)	22 (17.5)	15 (19.0)	7 (14.9)
**Vital status (6.2.2023), n (%)**			
Alive	114 (90.5)	73 (92.4)	41 (87.2)
Dead	12 (9.5)	6 (7.6)	6 (12.8)
**No somatic late effects follow-up, n (%)**	32 (25.4)	19 (24.1)	13 (27.7)
**Somatic late effects follow-up, n (%)**	94 (74.6)	60 (75.9)	34 (72.3)
**Follow-up time for somatic late effects (y), mean ± SD**	27.8 ± 7.2	27.0 ± 7.7	29.2 ± 6.1
**Number of somatic late effects, mean ± SD**	6.3 ± 4.5	5.8 ± 4.2	7.8 ± 5.1
**At least one somatic late effect, n (%)**	86 (91.5)	57 (95.0)	29 (85.3)
Reproductive organs, n (%)	0 (0.0)	0 (0.0)	0 (0.0)
Respiratory, n (%)	2 (2.1)	1 (1.7)	1 (2.9)
Renal and urinary, n (%)	3 (3.2)	2 (3.3)	1 (2.9)
Gastrointestinal and hepatobiliary, n (%)	6 (6.4)	4 (6.7)	2 (5.9)
Hematological, n (%)	7 (7.4)	1 (1.7)	6 (17.6)
Immunological and infectious, n (%)	8 (8.5)	3 (5.0)	5 (14.7)
Auditory, n (%)	19 (20.2)	11 (18.3)	8 (23.5)
Visual, n (%)	34 (36.2)	22 (36.7)	12 (35.3)
Cardiovascular, n (%)	37 (39.4)	24 (40.0)	13 (38.2)
Musculoskeletal and skin, n (%)	52 (55.3)	31 (51.7)	21 (61.8)
Endocrine, n (%)	54 (57.4)	29 (48.3)	25 (73.5)
Neurological and neurocognitive, n (%)	69 (73.4)	43 (71.7)	26 (76.5)
**Second primary cancers, n (%)**	30 (23.8)	15 (19.0)	12 (25.5)

## Data Availability

The raw data supporting the conclusions of this article will be made available by the authors on request. Individual patient data are not available. Requests to access the datasets should be directed to amihor@onko-i.si.

## Supplementary Materials

The following supporting information can be downloaded at https://www.mdpi.com/article/10.3390/cancers17040580/s1: Figure S1: Process of acquiring the final cohort of childhood and adolescent cancer survivors eligible for a late effects follow-up; Table S1: Basic characteristics and ranking of individual registry results, by questionnaire category and overall; Table S2: Classification of the late effects used in the exploratory study; Table S3: Cox hazard analyses for incidence of late effects by organ systems and second primary cancers; Table S4: Results of the online search for additional registries.

## Appendix A

**Questionnaire for ENCR members about childhood cancer registration** **practices** **and childhood cancer surveillance of late effects within cancer registries** **:**

Dear cancer registry colleagues!

We are conducting a short survey with the aim to better understand the level of detail of data on registered paediatric cancer cases in Europe. The main purpose is to identify registries that have or are planning to have detailed data on paediatric cancer patients and furthermore, if any registries have or are planning to have in place systematic registration of late effects of childhood cancer treatment for potential collaboration in the future.

We kindly ask you to fill out the questionnaire, which will take you about 5–10 min.

If you have any questions, please contact Ana Mihor at amihor@onko-i.si.

Thank you for your cooperation and kind regards,

Ana Mihor and Vesna Zadnik from the Slovenian Cancer Registry

1. Please, enter the cancer registry name
  _______ *(text, obligatory)*
2. Does your cancer registry include paediatric patients? *(not multiple choice, obligatory)*
  - Yes
  - No
    (a) If NO ---> 2.1 Can you provide name(s) of cancer registry(ies) that register paediatric patients in your country? _______ *(text, not obligatory)* ---> Thank you for your time.
    (b) If YES ---> *go to following questions*:
3. What is the extent of the registry coverage? *(not multiple choice, obligatory)*
  - National
  - Regional (Regional coverage refers to non-national coverage, for example: region, province, city, etc.) ---> 3.1 Please, specify the area covered by the cancer registry: ________ *(text, obligatory)*
4. What age range of childhood cancer cases is included in your registry? *(multiple choice, obligatory)*
  - 0–14 years
  - 0–19 years
  - Other ---> 4.1. Please, specify the range of age:__________ to age _________ *(numerical, obligatory)*
5. Which malignant cancer entities do you register? *(not multiple choice, obligatory)*
  - All
  - Specific entities only (such as solid tumours, haematological malignancies etc.) ---> 5.1. Please, specify:_________ *(text, obligatory)*
6. Do you also register non-malignant tumours? *(not multiple choice, obligatory)*
  • Yes
  • No
  • No, but we are planning to in the future
    (a) If no ---> 7.
    (b) If yes ---> 6.1
    (c) If no, but we are planning to in the future ---> 6.2
  6.1. Which non-malignant entities do you (or are planning to) register? (*table with several yes/no/we are planning to, obligatory)*
    - Central nervous system tumours (ICD-O-3: C70–C72)
    - Other intracranial tumours (pituitary, pineal, craniopharyngeal) (ICD-O-3: C75.1–C75.3)
    - Other tumours ---> 6.1.1. Please, specify __________ *(text, obligatory)*
  6.2. Which non-malignant tumours are you planning to register *(table with several yes/no, obligatory)*
    - Central nervous system tumours (ICD-O-3: C70–C72)
    - Other intracranial tumours (pituitary, pineal, craniopharyngeal) (ICD-O-3: C75.1–C75.3)
    - Other tumours ---> 6.2.1. Please, specify __________ *(text, obligatory)*
7. In addition to most basic data agreed for general/adult cancer registries (e.g., ENCR rules for coding), which detailed registry data do you or are planning to routinely collect on paediatric cancer patients (meaning the data can be extracted from the database with only basic manipulation for potential data calls)? *(tables with several yes/no/we are planning to, obligatory)*
  7.1. Diagnosis
    - Topography, morphology and behaviour for coding the tumour according to the International Childhood Cancer Classification, third edition (ICCC-3)
    - Specific diagnostic imaging tests performed (with or without further details), for example MRI, CT, PET scan, ultrasound, etc.
    - Other specific diagnostic procedures performed (with or without further details), for example tissue biopsy, bone marrow biopsy, cerebrospinal fluid examination.
  7.2. Staging
    - TNM stage (where applicable)
    - Toronto stage tier I
    - Toronto stage tier II
    - Other staging system ---> 7.2.1 Please, specify other staging system(s): ________ *(text, obligatory)*
    - Non-stage prognostic factors (i.e., Wingless/Sonic Hedgehog for medulloblastoma, FKHR-PAX3/7 rhabdomyosarcoma, N-myc for neuroblastoma etc.)
    - Topography of metastases
  7.3. Initial treatment of primary tumour
    - Surgery yes/no
    - Date of surgery
    - Type of surgery
    - Radiotherapy yes/no
    - Date of start of radiotherapy
    - Radiotherapy—body sections or organs irradiated
    - Radiotherapy—cumulative doses per body sections or organs
    - Systemic therapy yes/no
    - Date of start of systemic therapy
    - Chemotherapy yes/no
    - Specific chemotherapeutics received
    - Targeted therapy yes/no
    - Specific targeted therapy drugs received
    - Immunotherapy yes/no
    - Specific immunotherapy drugs received
    - Hormone therapy yes/no
    - Specific hormone therapy drugs received
    - Cumulative doses of specific therapeutics
    - Intrathecal applications of specific therapeutics
    - Stem cell and bone marrow transplantations yes/no
    - Stem cell and bone marrow transplantations—further details (e.g., type of transplant, therapy received, GVHD disease)
    - Other treatments received yes/no (with or without further details), for example solid organ transplantations, genetic treatments, fertility preservation
    - Acute side effects of treatments
  7.4. Disease progression
    - 7.4.1 Presence/absence of progression/relapse/recurrence *(if yes/planning to selected, text appears)* ---> 7.4.1.1
      ○ Please provide any comments on progression/relapse/recurrence definition that you use or are planning to use: _______ *(text, not obligatory)*
    - 7.4.2 Date of progression/relapse/recurrence
    - 7.4.3 Type of progression/relapse/recurrence
    - 7.4.4 Treatment of progression/relapse/recurrence *(if yes/planning to selected, dropdown menu, not multiple choice)* ---> 7.4.1.2
      ○ to same detail as primary treatment
      ○ to lesser detail as primary treatment
      ○ to greater detail as primary treatment
    - 7.4.5 Topography of metastases
  7.5. Follow-up
    - Vital status
    - Date of the last known vital status
    - Underlying cause of death *(if yes/planning to selected, text appears)* ---> *7.5.1 Please, specify the classification(s) used for coding the cause of death:*
      ○ Cause of death classification(s), please describe: _____________ *(text, obligatory)*
    - Multiple cause of death *(if yes/planning to selected, text appears)* ---> *7.5.2 Please, specify the classification(s) used for coding the cause of death:*
      ○ Cause of death classification(s), please describe: _____________ *(text, obligatory)*
  7.6. Other data
    - Previous primary cancers
    - Second primary cancers
    - Other ---> 7.6.1 Please, specify _______ *(text, obligatory)*
8. Do you or are you planning to routinely and systematically register late effects of childhood cancer treatment, other than secondary cancers (such as cardiovascular disease, endocrine dysfunction, cognitive deficits etc.)? *(not multiple choice, obligatory)*
  • Yes
  • No
  • No, but we are planning to
    (a) If NO ---> 8.1 Can you provide name(s) of cancer registries (or other institutions) that systematically register late effects in your country? _______ *(text, not obligatory)* ---> Thank you for your time.
    (b) If YES ---> 8.2, then 9.1, 10.1, 11.1, 12.1 and 13.1
    (c) If no, but we are planning to---> 8.3, then 9.2, 10.2, 11.2, 12.2 and 13.2
  8.2. From which incidence year did you start registering late effects? *(not multiple choice, obligatory)*
    - From incidence year _______ *(dropdown menu for year)*
    - I do not know
  8.3. From which incidence year will you start registering late effects? *(not multiple choice, obligatory)*
    - From incidence year _______ (dropdown menu for year)
    - I do not know yet
9 9.1. What is (are) the data source(s) for the collection of late effects data? *(multiple choice, obligatory)*
    - National/regional patient database
    - Hospital databases
    - Outpatient databases
    - Primary health care institutions
    - Other ---> 9.1.1 Please, specify _______ *(text, obligatory)*
  9.2. What will be the data source(s) for the collection of late effects data? (multiple choice, obligatory, if I do not know yet selected, then others not possible)
    - National/regional patient database
    - Hospital databases
    - Outpatient databases
    - Primary health care institutions
    - Other ---> 9.2.1 Please, specify _______ *(text, obligatory)*
    - I do not know yet
10 10.1. Which classification(s) of late effects do you use? *(multiple choice, obligatory)*
    - CTCAE (Common Terminology Criteria for Adverse Events)
    - Modified CTCAE
    - ICD-10
    - ICD-9
    - Other ---> 10.1.1 Please, specify _______ *(text, obligatory)*
  10.2. Which classification(s) of late effects will you use? (multiple choice, obligatory, if I do not know yet selected, then others not possible)
    - CTCAE (Common Terminology Criteria for Adverse Events)
    - Modified CTCAE
    - ICD-10
    - ICD-9
    - Other ---> 10.2.1 Please, specify _______ *(text, obligatory)*
    - I do not know yet
11. 11.1. Do you record the date of onset of late effects? *(not multiple choice, obligatory)*
    - Yes
    - No
  11.2. Will you record the date of onset of late effects? *(not multiple choice, obligatory)*
    - Yes
    - No
    - I do not know yet
12. 12.1. Do you record the grade/severity of late effects? *(not multiple choice, obligatory)*
    - Yes
    - No
  12.2. Will you record the grade/severity of late effects? *(not multiple choice, obligatory)*
    - Yes
    - No
    - I do not know yet
13. Can we contact you for further details on late effects registration practices? *(not multiple choice, obligatory)*
  - Yes
  - No
    (a) If YES ---> 13.1
    (b) If NO ---> 14
14. 14.1. Please, write your name and your email:
    Name: _______ (text, obligatory)
    E-mail: _______ (text, obligatory)
  14.2. Do you have any additional comments/suggestions? ___________________ *(text, not obligatory)*
    ---> Thank you for your time

## References

[1] Gatta G., Botta L., Rossi S., Aareleid T., Bielska-Lasota M., Clavel J., Dimitrova N., Jakab Z., Kaatsch P., Lacour B. (2014). Childhood Cancer Survival in Europe 1999–2007: Results of EUROCARE-5—A Population-Based Study. Lancet Oncol..

[2] Trama A., Botta L., Foschi R., Ferrari A., Stiller C., Desandes E., Maule M.M., Merletti F., Gatta G. (2016). Survival of European Adolescents and Young Adults Diagnosed with Cancer in 2000–07: Population-Based Data from EUROCARE-5. Lancet Oncol..

[3] Erdmann F., Frederiksen L.E., Bonaventure A., Mader L., Hasle H., Robison L.L., Winther J.F. (2021). Childhood Cancer: Survival, Treatment Modalities, Late Effects and Improvements over Time. Cancer Epidemiol..

[4] Steliarova-Foucher E., Fidler M.M., Colombet M., Lacour B., Kaatsch P., Piñeros M., Soerjomataram I., Bray F., Coebergh J.W., Peris-Bonet R. (2018). Changing Geographical Patterns and Trends in Cancer Incidence in Children and Adolescents in Europe, 1991–2010 (Automated Childhood Cancer Information System): A Population-Based Study. Lancet Oncol..

[5] Sharma R. (2021). A Systematic Examination of Burden of Childhood Cancers in 183 Countries: Estimates from GLOBOCAN 2018. Eur. J. Cancer Care.

[6] PDQ Pediatric Treatment Editorial Board (2020). Late Effects of Treatment for Childhood Cancer (PDQ(R)): Health Professional Version.

[7] Ness K.K., Kirkland J.L., Gramatges M.M., Wang Z., Kundu M., McCastlain K., Li-Harms X., Zhang J., Tchkonia T., Pluijm S.M.F. (2018). Premature Physiologic Aging as a Paradigm for Understanding Increased Risk of Adverse Health Across the Lifespan of Survivors of Childhood Cancer. J. Clin. Oncol..

[8] Cupit-Link M.C., Kirkland J.L., Ness K.K., Armstrong G.T., Tchkonia T., LeBrasseur N.K., Armenian S.H., Ruddy K.J., Hashmi S.K. (2017). Biology of Premature Ageing in Survivors of Cancer. ESMO Open.

[9] Winther J.F., Kenborg L., Byrne J., Hjorth L., Kaatsch P., Kremer L.C.M., Kuehni C.E., Auquier P., Michel G., de Vathaire F. (2015). Childhood Cancer Survivor Cohorts in Europe. Acta Oncol..

[10] Robison L.L., Mertens A.C., Boice J.D., Breslow N.E., Donaldson S.S., Green D.M., Li F.P., Meadows A.T., Mulvihill J.J., Neglia J.P. (2002). Study Design and Cohort Characteristics of the Childhood Cancer Survivor Study: A Multi-Institutional Collaborative Project. Med. Pediatr. Oncol..

[11] Hudson M.M., Ness K.K., Nolan V.G., Armstrong G.T., Green D.M., Morris E.B., Spunt S.L., Metzger M.L., Krull K.R., Klosky J.L. (2011). Prospective Medical Assessment of Adults Surviving Childhood Cancer: Study Design, Cohort Characteristics, and Feasibility of the St. Jude Lifetime Cohort Study. Pediatr. Blood Cancer.

[12] Children’s Oncology Group Long-Term Follow up Guidelines for Survivors of Childhood, Adolescent and Young Adult Cancers. http://www.survivorshipguidelines.org/pdf/2018/COG_LTFU_Guidelines_v5.pdf.

[13] van Kalsbeek R.J., van der Pal H.J.H., Kremer L.C.M., Bardi E., Brown M.C., Effeney R., Winther J.F., Follin C., den Hartogh J., Haupt R. (2021). European PanCareFollowUp Recommendations for Surveillance of Late Effects of Childhood, Adolescent, and Young Adult Cancer. Eur. J. Cancer.

[14] Mulder R.L., Kremer L.C.M., Hudson M.M., Bhatia S., Landier W., Levitt G., Constine L.S., Wallace W.H., van Leeuwen F.E., Ronckers C.M. (2013). Recommendations for Breast Cancer Surveillance for Female Survivors of Childhood, Adolescent, and Young Adult Cancer given Chest Radiation: A Report from the International Late Effects of Childhood Cancer Guideline Harmonization Group. Lancet Oncol..

[15] Armenian S.H., Hudson M.M., Mulder R.L., Chen M.H., Constine L.S., Dwyer M., Nathan P.C., Tissing W.J.E., Shankar S., Sieswerda E. (2015). Recommendations for Cardiomyopathy Surveillance for Survivors of Childhood Cancer: A Report from the International Late Effects of Childhood Cancer Guideline Harmonization Group. Lancet Oncol..

[16] Clemens E., van den Heuvel-Eibrink M.M., Mulder R.L., Kremer L.C.M., Hudson M.M., Skinner R., Constine L.S., Bass J.K., Kuehni C.E., Langer T. (2019). Recommendations for Ototoxicity Surveillance for Childhood, Adolescent, and Young Adult Cancer Survivors: A Report from the International Late Effects of Childhood Cancer Guideline Harmonization Group in Collaboration with the PanCare Consortium. Lancet Oncol..

[17] van Dorp W., Mulder R.L., Kremer L.C.M., Hudson M.M., van den Heuvel-Eibrink M.M., van den Berg M.H., Levine J.M., van Dulmen-den Broeder E., di Iorgi N., Albanese A. (2016). Recommendations for Premature Ovarian Insufficiency Surveillance for Female Survivors of Childhood, Adolescent, and Young Adult Cancer: A Report From the International Late Effects of Childhood Cancer Guideline Harmonization Group in Collaboration With Th. J. Clin. Oncol..

[18] Skinner R., Mulder R.L., Kremer L.C., Hudson M.M., Constine L.S., Bardi E., Boekhout A., Borgmann-Staudt A., Brown M.C., Cohn R. (2017). Recommendations for Gonadotoxicity Surveillance in Male Childhood, Adolescent, and Young Adult Cancer Survivors: A Report from the International Late Effects of Childhood Cancer Guideline Harmonization Group in Collaboration with the PanCareSurFup Consort. Lancet Oncol..

[19] Clement S.C., Kremer L.C.M., Verburg F.A., Simmons J.H., Goldfarb M., Peeters R.P., Alexander E.K., Bardi E., Brignardello E., Constine L.S. (2018). Balancing the Benefits and Harms of Thyroid Cancer Surveillance in Survivors of Childhood, Adolescent and Young Adult Cancer: Recommendations from the International Late Effects of Childhood Cancer Guideline Harmonization Group in Collaboration with the P. Cancer Treat. Rev..

[20] Grosclaude P., Zadnik V., Launoy G., Zadnik V., Coleman M.P. (2021). Population-Based Cancer Registries: A Data Stream to Help Build an Evidence-Based Cancer Policy for Europe and for European Countries. Social Environment and Cancer in Europe: Towards an Evidence-Based Public Health Policy.

[21] European Network of Cancer Registries (ENCR). https://www.encr.eu/.

[22] Survivorship Passport|A Project to Provide Every European Childhood Cancer Survivor a Document, Paper and Electronic Based, Containing Cancer History and Therapy Information. http://www.survivorshippassport.org/.

[23] Chronaki C., Charalambous E., Cangioli G., Schreier G., van den Oever S., van der Pal H., Kremer L., Uyttebroeck A., Van den Bosch B., Trinkunas J. (2022). Factors Influencing Implementation of the Survivorship Passport: The IT Perspective. Stud. Health. Technol. Inform..

[24] Hudson M.M., Ehrhardt M.J., Bhakta N., Baassiri M., Eissa H., Chemaitilly W., Green D.M., Mulrooney D.A., Armstrong G.T., Brinkman T.M. (2017). Approach for Classification and Severity Grading of Long-Term and Late-Onset Health Events among Childhood Cancer Survivors in the St. Jude Lifetime Cohort. Cancer Epidemiol Biomark. Prev..

[25] World Health Organization (2013). International Classification of Diseases for Oncology (ICD-O).

[26] Steliarova-Foucher E., Stiller C., Lacour B., Kaatsch P. (2005). International Classification of Childhood Cancer, Third Edition. Cancer.

[27] International Agency for Research on Cancer Cancer Risk in Childhood Cancer Survivors (CRICCS). https://criccs.iarc.who.int/.

[28] Gupta S., Aitken J.F., Bartels U., Brierley J., Dolendo M., Friedrich P., Fuentes-Alabi S., Garrido C.P., Gatta G., Gospodarowicz M. (2016). Paediatric Cancer Stage in Population-Based Cancer Registries: The Toronto Consensus Principles and Guidelines. Lancet Oncol..

[29] Botta L., Gatta G., Didonè F., Cortes A.L., Pritchard-Jones K., The BENCHISTA Project Working Group (2022). International Benchmarking of Childhood Cancer Survival by Stage at Diagnosis: The BENCHISTA Project Protocol. PLoS ONE.

[30] Michel G., von der Weid N.X., Zwahlen M., Adam M., Rebholz C.E., Kuehni C.E. (2007). Swiss Childhood Cancer Registry; Swiss Paediatric Oncology Group (SPOG) Scientific Committee The Swiss Childhood Cancer Registry: Rationale, Organisation and Results for the Years 2001–2005. Swiss Med. Wkly..

[31] Curry H.L., Parkes S.E., Powell J.E., Mann J.R. (2006). Caring for Survivors of Childhood Cancers: The Size of the Problem. Eur. J. Cancer.

[32] Poulalhon C., Vignon L., Idbrik L., Bernier-Chastagner V., Fabre M., Schleiermacher G., Dijoud F., Perrin C., Varlet P., Faure L. (2020). Data Resource Profile: The French Childhood Cancer Observation Platform (CCOP). Int. J. Epidemiol..

[33] Erdmann F., Kaatsch P., Grabow D., Spix C. (2020). German Childhood Cancer Registry—Annual Report 2019 (1980–2018).

[34] Kaatsch P., Trübenbach C., Kaiser M., Erdmann F., Spix C., Grabow D. (2022). [The 41,000 long-term survivor cohort of the German Childhood Cancer Registry]. Bundesgesundheitsblatt Gesundheitsforschung Gesundheitsschutz.

[35] Nationwide Registry for Childhood Hematological Malignancies and Solid Tumors (NARECHEM-ST). http://www.narechem.gr/node/1.

[36] Borgulya G., Jakab Z., Schuler D., Garami M. (2004). Establishing an Internet-Based Paediatric Cancer Registration and Communication System for the Hungarian Paediatric Oncology Network. Stud. Health Technol. Inform..

[37] Cromie K.J., Crump P., Hughes N.F., Milner S., Greenfield D., Jenkins A., McNally R., Stark D., Stiller C.A., Glaser A.W. (2022). Data Resource Profile: Yorkshire Specialist Register of Cancer in Children and Young People (Yorkshire Register). Int. J. Epidemiol..

[38] Spanish Registry of Childhood Tumors RETI-SEHOP. https://www.uv.es/rnti/index.html.

[39] Petersson-Ahrholt M., Wiebe T., Hjorth L., Relander T., Linge H.M. (2019). Development and Implementation of Survivorship Tools to Enable Medical Follow-Up After Childhood Cancer Treatment in Southern Sweden. JCO Clin. Cancer Inform..

[40] Wiebe T., Hjorth L., Marotta Kelly M., Linge H.M., Garwicz S. (2018). A Population Based Pediatric Oncology Registry in Southern Sweden: The BORISS Registry. Eur. J. Epidemiol..

[41] Jaspers M., van den Bos C., Caron H. (2001). A National Late Cancer Treatment Effects Registry in the Netherlands: LATER. Proc. AMIA Symp..

[42] Feijen E.A.M., Teepen J.C., van Dulmen-den Broeder E., van den Heuvel-Eibrink M.M., van der Heiden-van der Loo M., van der Pal H.J.H., de Vries A.C.H., Louwerens M., Bresters D., Versluys B. (2023). Clinical Evaluation of Late Outcomes in Dutch Childhood Cancer Survivors: Methodology of the DCCSS LATER 2 Study. Pediatr. Blood Cancer.

[43] Otth M., Drozdov D., Hügli C., Scheinemann K. (2021). Young Survivors at KSA: Registry for Standardised Assessment of Long-Term and Late-Onset Health Events in Survivors of Childhood and Adolescent Cancer—A Study Protocol. BMJ Open.

[44] Ferrari A., Stark D., Peccatori F.A., Fern L., Laurence V., Gaspar N., Bozovic-Spasojevic I., Smith O., De Munter J., Derwich K. (2021). Adolescents and Young Adults (AYA) with Cancer: A Position Paper from the AYA Working Group of the European Society for Medical Oncology (ESMO) and the European Society for Paediatric Oncology (SIOPE). ESMO Open.

[45] European Network of Cancer Registries European Network of Cancer Registries Working Groups. https://encr.eu/Activities/Working-groups.

[46] Argyriadi E.A., Steffen I.G., Chen-Santel C., Lissat A., Attarbaschi A., Bourquin J.P., Henze G., von Stackelberg A. (2024). Prognostic Relevance of Treatment Deviations in Children with Relapsed Acute Lymphoblastic Leukemia who were Treated in the ALL-REZ BFM 2002 Study. Leukemia.

[47] Streefkerk N., Fioole L.C.E., Beijer J.G.M., Feijen E.A.M., Teepen J.C., Winther J.F., Ronckers C.M., Loonen J.J., van Dulmen-den Broeder E., Skinner R. (2020). Large Variation in Assessment and Outcome Definitions to Describe the Burden of Long-Term Morbidity in Childhood Cancer Survivors: A Systematic Review. Pediatr. Blood Cancer.

[48] Otth M., Drozdov D., Scheinemann K. (2022). Feasibility of a Registry for Standardized Assessment of Long-Term and Late-Onset Health Events in Survivors of Childhood and Adolescent Cancer. Sci. Rep..

[49] Mihor A., Martos C., Giusti F., Zadravec-Zaletel L., Tomšič S., Lokar K., Žagar T., Birk M., Bric N., Zadnik V. Survey of Childhood and Adolescent Cancer Registration in Europe and Example of Slovenia. Proceedings of the ENCR IACR Scientific Conference.

[50] Roddy E., Mueller S. (2016). Late Effects of Treatment of Pediatric Central Nervous System Tumors. J. Child Neurol..

[51] Česen Mazić M., Reulen R.C., Jazbec J., Zadravec Zaletel L. (2022). Trends in Treatment of Childhood Cancer and Subsequent Primary Neoplasm Risk. Radiol. Oncol..

[52] Jereb B., Korenjak R., Kržišnik C., Petrič-Grabnar G., Zadravec-Zaletel L., Anzic J., Stare J. (1994). Late Sequelae in Children Treated for Brain Tumors and Luekemia. Acta Oncol..

[53] Macedoni-Lukšič M., Jereb B., Todorovski L. (2003). Long-Term Sequelae in Children Treated for Brain Tumors: Impairments, Disability, and Handicap. Pediatr. Hematol. Oncol..

[54] Surveillance for Subsequent Neoplasms of the CNS for Childhood, Adolescent, and Young Adult Cancer Survivors: A Systematic Review and Recommendations from the International Late Effects of Childhood Cancer Guideline Harmonization Group—The Lancet Oncology. https://www.thelancet.com/journals/lanonc/article/PIIS1470-2045.

[55] Jensen M.V., Rugbjerg K., de Fine Licht S., Johansen C., Schmiegelow K., Andersen K.K., Winther J.F. (2018). Endocrine Late Effects in Survivors of Cancer in Adolescence and Young Adulthood: A Danish Population-Based Cohort Study. JAMA Netw. Open.

[56] van der Heide I., Moye Holz D., Rijken M., Hansen J. (2022). Access to Financial Products for Persons with a History of Cancer in EU Member States. An Exploratory Study.

[57] Forjaz G., Kohler B., Coleman M.P., Steliarova-Foucher E., Negoita S., Guidry Auvil J.M., Michels F.S., Goderre J., Wiggins C., Durbin E.B. (2025). Making the Case for an International Childhood Cancer Data Partnership. J. Natl. Cancer Inst..

