# Supplementary figures and images for: The Population-Level Surveillance of Childhood and Adolescent Cancer and Its Late Effects in Europe with an Example of an Effective System at the Slovenian Cancer Registry
Source: Cancers (Basel). 2025 Feb 8;17(4):580. doi: 10.3390/cancers17040580 (PMC11853519; doi:10.3390/cancers17040580)

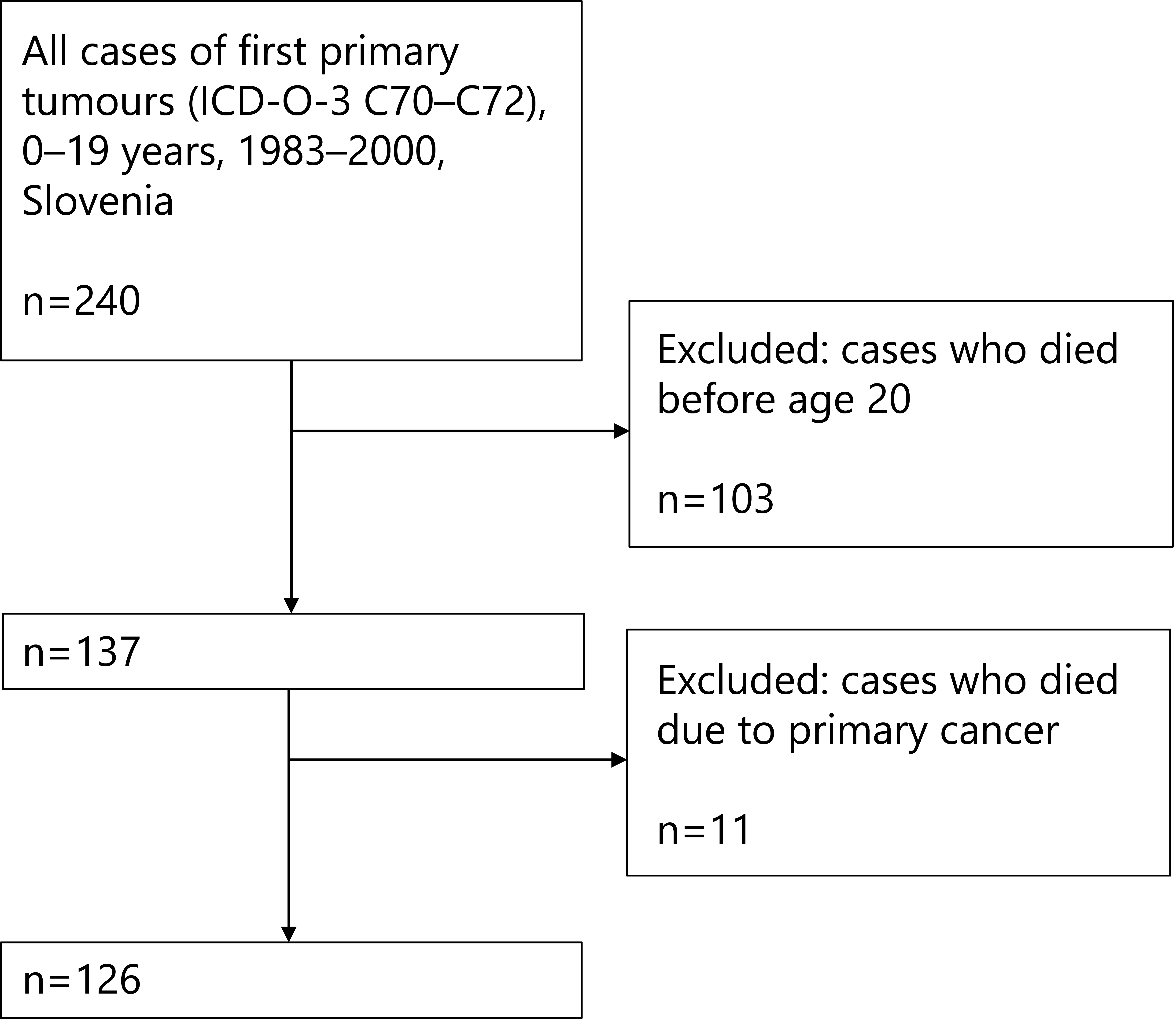

Supplement: Supplementary file 1 [file cancers-17-00580-s001.zip › MihorA_Cancers_supplementary Figure S1_corrected_submitted.tif]
